# On the origin of *Mycobacterium ulcerans,* the causative agent of Buruli ulcer

**DOI:** 10.1186/1471-2164-13-258

**Published:** 2012-06-19

**Authors:** Kenneth D Doig, Kathryn E Holt, Janet AM Fyfe, Caroline J Lavender, Miriam Eddyani, Françoise Portaels, Dorothy Yeboah-Manu, Gerd Pluschke, Torsten Seemann, Timothy P Stinear

**Affiliations:** 1 Department of Microbiology and Immunology, University of Melbourne, Parkville, Australia; 2 Victorian Bioinformatics Consortium, Monash University, Clayton, Australia; 3 Victorian Infectious Diseases Reference Laboratory, North Melbourne, Australia; 4 Department of Biomedical Sciences, Institute of Tropical Medicine, Antwerp, Belgium; 5 Noguchi Memorial Institute for Medical Research, University of Ghana, Legon, Ghana; 6 Swiss Tropical and Public Health Institute, Molecular Immunology, Basel, Switzerland; 7 University of Basel, Basel, Switzerland

## Abstract

**Background:**

*Mycobacterium ulcerans* is an unusual bacterial pathogen with elusive origins. While closely related to the aquatic dwelling *M. marinum*, *M. ulcerans* has evolved the ability to produce the immunosuppressive polyketide toxin mycolactone and cause the neglected tropical disease Buruli ulcer. Other mycolactone-producing mycobacteria (MPM) have been identified in fish and frogs and given distinct species designations (*M. pseudoshottsii, M. shinshuense*, *M. liflandii* and *M. marinum*), however the evolution of *M. ulcerans* and its relationship to other MPM has not been defined. Here we report the comparative analysis of whole genome sequences from 30 MPM and five *M. marinum*.

**Results:**

A high-resolution phylogeny based on genome-wide single nucleotide polymorphisms (SNPs) showed that *M. ulcerans* and all other MPM represent a single clonal group that evolved from a common *M. marinum* progenitor. The emergence of the MPM was driven by the acquisition of the pMUM plasmid encoding genes for the biosynthesis of mycolactones. This change was accompanied by the loss of at least 185 genes, with a significant overrepresentation of genes associated with cell wall functions. Cell wall associated genes also showed evidence of substantial adaptive selection, suggesting cell wall remodeling has been critical for the survival of MPM. Fine-grain analysis of the MPM complex revealed at least three distinct lineages, one of which comprised a highly clonal group, responsible for Buruli ulcer in Africa and Australia. This indicates relatively recent transfer of *M. ulcerans* between these continents, which represent the vast majority of the global Buruli ulcer burden. Our data provide SNPs and gene sequences that can differentiate *M. ulcerans* lineages, suitable for use in the diagnosis and surveillance of Buruli ulcer.

**Conclusions:**

*M. ulcerans* and all mycolactone-producing mycobacteria are specialized variants of a common *Mycobacterium marinum* progenitor that have adapted to live in restricted environments. Examination of genes lost or retained and now under selective pressure suggests these environments might be aerobic, and extracellular, where slow growth, production of an immune suppressor, cell wall remodeling, loss or modification of cell wall antigens, and biofilm-forming ability provide a survival advantage. These insights will guide our efforts to find the elusive reservoir(s) of *M. ulcerans* and to understand transmission of Buruli ulcer.

## Background

The genomes of *Mycobacterium ulcerans* and *Mycobacterium marinum* are closely related, sharing >97% overall nucleotide identity [1], but cause very different kinds of infections in humans. *M. marinum* causes minor skin infections, characterised by intracellular bacteria and the granulomatous lesions that are features of infection with many mycobacterial pathogens, notably *Mycobacterium tuberculosis*[2]. In contrast, *M. ulcerans* causes Buruli ulcer (BU), a slowly progressing, ulcerative disease characterized by necrosis of subcutaneous tissue. BU has a characteristic histopathology with large numbers of extracellular bacteria during the acute phase of the infection, with a marked lack of inflammatory response in advanced infection. This unusual pathology is principally mediated by an immunosuppressive polyketide called mycolactone, which is not produced by *M. marinum* or *M. tuberculosis*. In BU patients, mycolactone is present in cutaneous lesions but also diffuses and can be detected in serum [3,4].

Two main features differentiate the genomes of *M. ulcerans* and *M. marinum*. The first is the pMUM megaplasmid, found in *M. ulcerans* but absent from *M. marinum*[5,6]. This plasmid harbours three large genes (*mlsA1*: 51 kb, *mlsA2*: 7.2 kb, *mlsB*: 43 kb) that encode the polyketide synthases (PKSs) required for mycolactone synthesis [2]. The second is the insertion sequence (IS) IS*2404* that is absent from *M. marinum* but present in high copy number (>200) in *M. ulcerans* genomes. IS*2404* expansion in the *M. ulcerans* genome has led to the inactivation of many genes through disruption of coding and promoter sequences and has mediated the deletion of approximately 1 Mbp of DNA from *M. ulcerans* compared with *M. marinum*[1]. Together with loss of DNA, there is also evidence of extensive loss of gene function in *M. ulcerans -* the genome of *M. ulcerans* isolate Agy99 harbours 771 pseudogenes (inactivated genes), while the *M. marinum* genome harbours only 65. Acquisition of foreign DNA, IS expansion, pseudogene accumulation and genome reduction are features in common with bacterial populations that have passed through an evolutionary bottleneck [7-12], suggesting there has been constriction of population size during adaptation to a new, niche environment. Analysis of the *M. ulcerans* Agy99 genome showed deletion or inactivation of genes expressing potent T-cell antigens, and genes required for pigment biosynthesis, anaerobiosis, and intracellular growth [1]. This profile suggested a bacterium that has adapted to a dark, extracellular environment where slow growth, loss of immunogens and production of an immunosuppressive molecule provide a selective advantage [1,13]. In contrast, its progenitor, *M. marinum,* has the characteristics of both a specialist bacterium that can persist within an intracellular environment as well as a generalist that can survive in extracellular conditions. A niche environment for *M. ulcerans* has not yet been demonstrated although the recent discovery that Australian native possums inhabiting BU endemic areas appear to harbour the bacteria in their gastrointestinal tracts raises some interesting possibilities [14].

The species definition of *M. ulcerans* has recently been challenged by the discovery of variously named mycobacteria that also make mycolactones but are not always associated with BU. These mycobacteria, isolated from humans, fish and frogs in diverse geographic locations (including Japan, the Mediterranean, Israel, Belgium and the United States), have been variously named *Mycobacterium shinshuense, Mycobacterium marinumMycobacterium pseudoshottsii*, and “*Mycobacterium liflandii”*, the latter an unofficial species name with no standing in nomenclature [15-19]. Studies of these isolates using an 8-gene multi locus sequence typing (MLST) scheme and patterns of genome deletion suggest that they evolved from a *M. marinum* common ancestor and subsequently diverged into two principal lineages [20,21]. These mycolactone-producing *Mycobacteria* (MPM) lineages have been termed “classical” and “ancestral”. The “classical” MPM lineage includes *M. ulcerans* isolates associated with BU from Australia, South East Asia and Africa while the “ancestral” lineage includes the fish and frog isolates as well as *M. ulcerans* BU isolates from Japan, China, Mexico, Surinam and French Guiana [22,23]. We recently proposed that based on their genetic coherence all MPM should be renamed *M. ulcerans*, a proposition we will revisit in this paper [24].

Comparative genomic analysis of closely related bacteria has facilitated dramatic improvements in our understanding of bacterial pathogen evolution [25]. Low-coverage 454 and Illumina sequencing of three *M. ulcerans* isolates from Ghana and a single isolate from Japan demonstrated the capacity of high throughput sequencing to differentiate *M. ulcerans* co-circulating in a geographic region [26]. Here we report the sequencing and analysis of whole genomes from a diversity of isolates, which are representative of what we define as the *M. ulceransM. marinum* complex (abbreviated hereafter as MuMC). That is, mycobacteria previously identified as *M. marinumM. ulcerans* and other MPM that share >97% nucleotide identity based on MLST [23,24]. This collection includes mycobacteria isolated from humans, possums, a bilby (small Australian mammal), fish, frogs, an insect and an armadillo from diverse geographic locations. We used whole genome comparisons to reveal the phylogenomic relationship among these mycobacteria, enabling us to investigate the hypothesis that all MPM (including *M. ulcerans*) are derived from a common ancestor. Our genome comparisons strongly supported this hypothesis and showed that the niche-adapted genomic signature of *M. ulcerans* caused by reductive evolution was established in the progenitor of all MPM before their intercontinental dispersal. We also identify DNA segments that could be used to develop molecular diagnostic tools to distinguish MPM causing BU from other members of the MuMC.

## Results

### Isolate selection and sequence analysis strategy

**Figure 1:**
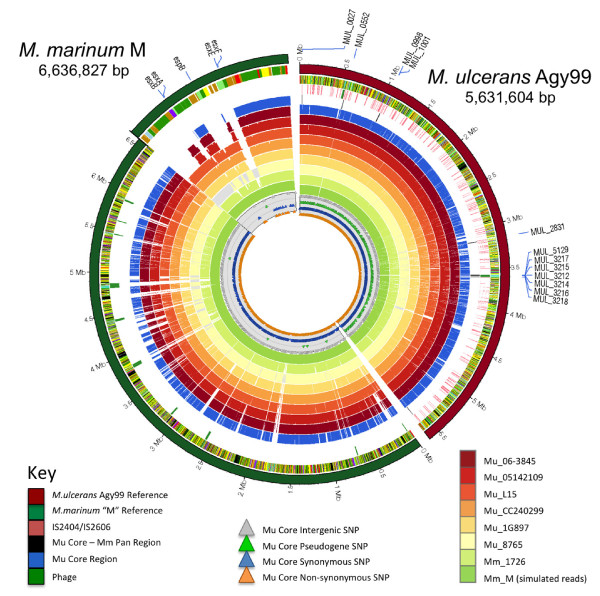
**Comparative genome content of selected study isolates to references *M. marinum* and *M. ulcerans.* ** Circos [36] plot of *de novo* assembled contigs, for a representative sample of the study isolates, mapped against the reference genomes of *M. marinum* M and *M. ulcerans* Agy99*.* The key below the figure describes the content. Moving inwards, the tracks are genes coloured by functional group (lipid metabolism – black; insertion seqs – aqua; others – green). The next track marks insertion sequences in pink. The remaining tracks show selected isolates and their coverage when de novo contigs are mapped against the reference. The isolates moving inwards are: Mu_06-3845 (Benin), Mu_05142109 (Australia), Mu_L15 (USA), Mu_CC240299 (Israel), Mu_1G897 (French Guiana), Mu_8765 (Japan), Mm_1726 (USA) and *M. marinum* “M”. The SNPs present in the *M.ulcerans* core genome are marked by type with coloured triangles in the innermost rings. The 12 loci overlapping the core *M. ulcerans* – pan *M. marinum* regions are shown with the outer most labels MUL_nnnn. The *M. marinum* region spanning the Esx-1 locus is scaled up x30 to highlight how it has been successively affected by deletion and is no longer part of the core *M. ulcerans* genome (blue circle).

**Figure 2:**
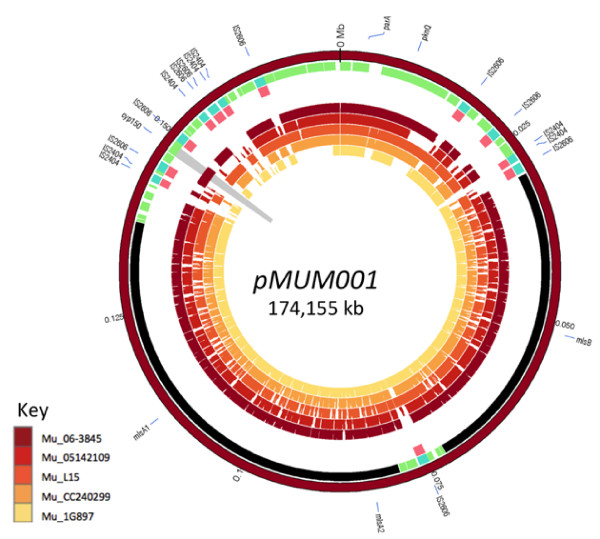
**Comparative genome content of plasmid pMUM001.** Circos [36] plot of de novo assembled contigs, for a sample of the study isolates, mapped against reference chromosome plasmid pMUM001. ISs and known genes are labelled in the outermost ring. Moving inwards, the tracks are genes coloured by functional group (lipid metabolism – black, insertion seqs – aqua, others – green). The next track marks insertion sequences in pink. The remaining tracks show selected isolates and their coverage when *de novo* contigs are mapped against the reference. The isolates moving inwards are: Mu_06-3845 (Benin), Mu_05142109 (Australia), Mu_L15 (USA), Mu_CC240299 (Israel), Mu_1G897 (French Guiana). The cyp150 gene (mup053) has been highlighted (grey wedge) to show its presence in African isolates and absence in other isolates.

In order to capture as much diversity as possible within the MuMC and minimise phylogenetic discovery bias [27], we selected 35 mycobacteria isolates from diverse members of the MuMC for whole genome sequencing and the comparison. These isolates were recovered from a range of host organisms including humans, possums, fish, frogs, an insect and an armadillo and include representatives of all the major MuMC sequence types defined by MLST [21]. The majority of isolates belong to the two dominant human pathogenic clades of MPM, namely *M. ulcerans* from Africa and Australia, however MPMs that have been given various species names such as *M. marinumM. pseudoshottsii* and *M. liflandii* were also represented. One genome was sequenced using Ion Torrent technology (single ended sequencing) and the remainder using Illumina GAIIx paired-end sequencing (Table 1 and Additional file 1: Table S1). For each isolate, genes were identified and annotated within *de novo* assembled contigs and then subjected to ortholog clustering by homology searches. Reads and contigs were aligned to both the *M. marinum* M and *M. ulcerans* Agy99 reference chromosomes (Figure 1) and the *M. ulcerans* Agy99 pMUM001 plasmid (Figure 2).

**Table 1:** Isolates used in this study

**Identifier**	**Alternate identifier**	**Origin**	**Source**	**Year isolated**	**Reference**
** *M. ulcerans* isolates**					
Mu_06-3845	ITM001441	Houedja, Ouinhi, Zou, Benin	Aquatic insect	2000	[28]
Mu_06-3846	ITM971116	Lalo, Couffo, Benin	Human	1997	This study
Mu_07-1082	ITM030216	Adjohoun, Ouémé, Benin	Human	2003	This study
Mu_1G897		Cayenne, French Guiana	Human	1988	[29]
Mu_Agy99		Ga District, Ghana	Human	1999	[1]
Mu_NM14.01		Ga District, Ghana	Human	2001	[30]
Mu_NM33.04		Amansie West District, Ghana	Human	2004	[30]
Mu_NM43.02		Ga District, Ghana	Human	2002	[30]
Mu_NM49.02		Ga District, Ghana	Human	2002	[30]
Mu_NM54.02		Ga District, Ghana	Human	2002	[30]
*Mu_DL045 (*M. marinum*)		Greece	Fish	2002	[19]
Mu_001506	ITM001506	Wokon, Ouinhi, Zou, Benin	Human	2000	This study
Mu_980535	ITM980535	Djigbé, Zé, Atlantique, Benin	Human	1998	This study
Mu_000945	ITM000945	Hwegoudo, Zé, Atlantique, Benin	Human	2000	This study
Mu_991845	ITM991845	Sagon, Ouinhi, Zou, Benin	Human	1999	This study
*Mu_CC240299 (*M. marinum*)		Israel	Fish	1999	[19]
*Mu_06-3844 (*M. marinum*)	ITM063844	Belgium	Fish	2006	[31]
*Mu_8765 (*M. shinshuense*)	ITM8765	Japan	Human	1980	[32]
*Mu_JKD8071 (*M. liflandii*)	128FXT	USA	Frog	2004	[33]
*Mu_L15 (*M. pseudoshottsii*)	L15	USA	Fish	2004	[17]
Mu_13822-70		Queensland, Australia	Human	1971	[34]
Mu_113	05152838	Point Lonsdale, Australia	Human	2005	This study
Mu_119	05159089	Frankston, Australia	Human	2005	This study
Mu_74	04140710	Point Lonsdale, Australia	Human	2004	This study
Mu_81	04149669	Point Lonsdale, Australia	Human	2004	This study
Mu_93	05131622	St Leonards, Australia	Human	2005	This study
Mu_05142109		East Gippsland, Australia	Possum	2005	This study
Mu_JKD8170		Point Lonsdale, Australia	Possum	2008	This study
Mu_08009899		Point Lonsdale, Australia	Human	2008	This study
Mu_JKD8049		Point Lonsdale, Australia	Human	2004	This study
** *M. marinum* isolates**					
Mm_99/89		NSW, Australia	Human	1994	[34]
Mm_99/84		Western Australia	Bilby (*Macrotis lagotis*)	1999	[34]
Mm_99/87		Western Australia	Human	1996	[34]
Mm_1726		Louisiana, USA	Armadillo	1986	[35]

*These isolates have been referred to by other species names as indicated but here are considered *M. ulcerans* and have been given the prefix “Mu_”. Note: Additional sequencing and assembly data in Additional file 1: Table S1.

**Figure 3:**
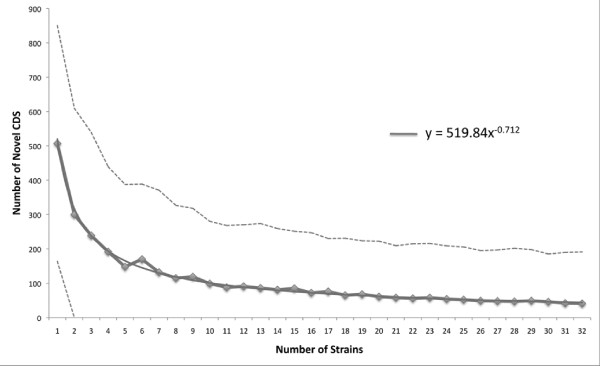
**Open genome analysis.** Median number of novel genes added per isolate (solid line) and the fitted power law model (equation inset). The median is for 1,000 random orderings of 32 of the study isolates. The dashed line shows the +/−1 standard deviation from the median.

To assess how well our collection represents the genetic diversity of the MuMC, we modelled the number of novel genes discovered with the addition of each new genome using a *de novo* assembly and an ortholog clustering approach (see Methods). We assessed the trend by fitting a power curve to estimate the exponent, which is indicative of whether the pan genome is ‘open’ or ‘closed’ (y = 519.8 x^-0.712^, R² = 0.99765) [37,38]. Our MuMC isolates indicated an open pan-genome (exponent > −1), meaning that the total number of genes would continue to increase if more isolates were included (Figure 3). Based on the model, we predict that further sequencing within the MuMC would, on average, reveal fewer than 42 new coding sequences (CDS) per additional isolate. The core set of conserved orthologous clusters of CDS for our study isolates was found to be 3,305 CDS and modelling predicted that additional sequencing would result in no further reduction in core genome size.

### Mycolactone-producing mycobacteria form a monophyletic group

The most sensitive method to identify a core set of conserved nucleotide positions among a set of genomes is via read mapping to a reference genome. The major advantages of this approach, compared to *de novo* assembly and ortholog clustering, are its independence from gene annotation and orthology and high (single nucleotide) resolution. Hence we mapped sequencing reads from each isolate to the *M. marinum* M reference and defined the MuMC core genome as the set of *M. marinum* M nucleotide positions that were covered by at least three reads from every isolate. Repeat regions and regions spanning PE/PPE genes were excluded from this analysis due to ambiguous read mapping and poor coverage, respectively. We found a core of 4,362,138 bp (65.7%) and 3,318 CDS of *M. marinum* M were present in all 35 mycobacteria of the complex.

**Figure 4:**
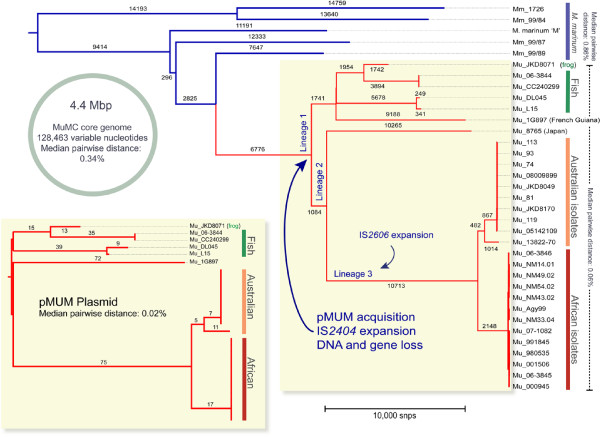
**MuMC phylogenomic analysis.** Neighbour joining dendrogram based on 128,463 variable common nucleotide positions across 34 sequenced isolates produced by SplitsTree4 [41] using uncorrected ‘P’ distances. The tree was rooted using *M. tuberculosis* as an outgroup. The major clustering of isolates are *M. marinum* isolates (4) – blue; Fish and frog isolates (5) – green; Japanese isolate (1) – Mu_8765; French Guiana isolate – Mu_1G897; Australian isolates (10) – orange; African isolates (13) – red. The scale bars show the median pairwise divergence for the set of isolates they span. The isolates that produce mycolactone are highlighted in pale yellow. The inset shows the pMUM plasmid SNP tree of 315 SNPs with edge lengths and a topology matching the corresponding isolates in the main tree. The details of the isolates can be found in Table 1.

Within this core genome, 128,463 variable nucleotide positions were identified among the sequenced MuMC genomes. These were randomly distributed around the chromosome (Figure 1) and were used to infer a distance-based neighbour joining phylogenetic tree (Figure 4). The tree topology confirms the previously inferred relationships between isolates by the lower-resolution MLST approach [21] and indicates that all mycolactone producing isolates (i.e. the MPM) belong to a single clonal group that diverged from a common *M. marinum* progenitor (Figure 4, root of red tree). In keeping with this observation, the MPM were relatively homogeneous with 0.06% median genome nucleotide divergence (interquartile range 0–0.37%) compared with *M. marinum* isolates with 0.86% nucleotide divergence (0.59 %-0.87%) (Figure 4). This corresponds with genome-wide average nucleotide identity of 97% among all genome pairs [39]. Within the MPM, at least three deep branching lineages were observed comprising, lineage 1: one human isolate from South America and globally distributed fish and frog isolates; lineage 2: a single *M. ulcerans* human isolate from Japan; and lineage 3: *M. ulcerans* human and other animal isolates from Africa and Australia. Lineage 3 corresponds to the previously reported “classical” MPM lineage, while the “ancestral” MPM lineage is refined here by lineages 1 and 2 [20]. The confirmation of a close genetic relationship between BU-causing MPM isolates from Africa and Australia (4,511 SNP differences) suggests that findings from studies of BU transmission in Australia may find corollaries in African BU endemic settings. Interestingly, there were even fewer nucleotide differences between some of the Lineage 1 isolates. For example *M. pseudoshottsii* and Mu_DL045, isolated two years apart from fish in the USA and Greece respectively, were separated by only 590 SNPs. Similarly, Mu_CC240299 and Mu_06-3844, isolated seven years and thousands of kilometres apart in Israel and Belgium, were separated by only 40 SNPs. These data indicate that clones of these mycolactone-producing ectotherm-infecting mycobacteria are circulating worldwide. We also calculated the proportion of *M. marinum* and *M. ulcerans* reference genome sequence covered by each isolate of the MuMC (Additional file 2: Figure S2). The plot shows that MPM isolates are clearly differentiated from *M. marinum* by their gene content, with less than 90% of genomic sequence conserved between MPM and *M. marinum* isolates. In contrast to other pathogens such as *Shigella,* which has four main variants that have emerged independently [40], it appears that MPM have evolved only once through the acquisition of the virulence plasmid pMUM and IS expansion. Hence our data confirm both high genomic coherence and a common ancestry among all the MPM, supporting our previous argument that all MPM should be considered *M. ulcerans*[24]. Hereafter we refer to all MPM as *M. ulcerans*.

### Geographical restriction of *M. ulcerans* over short time scales

**Figure 5:**
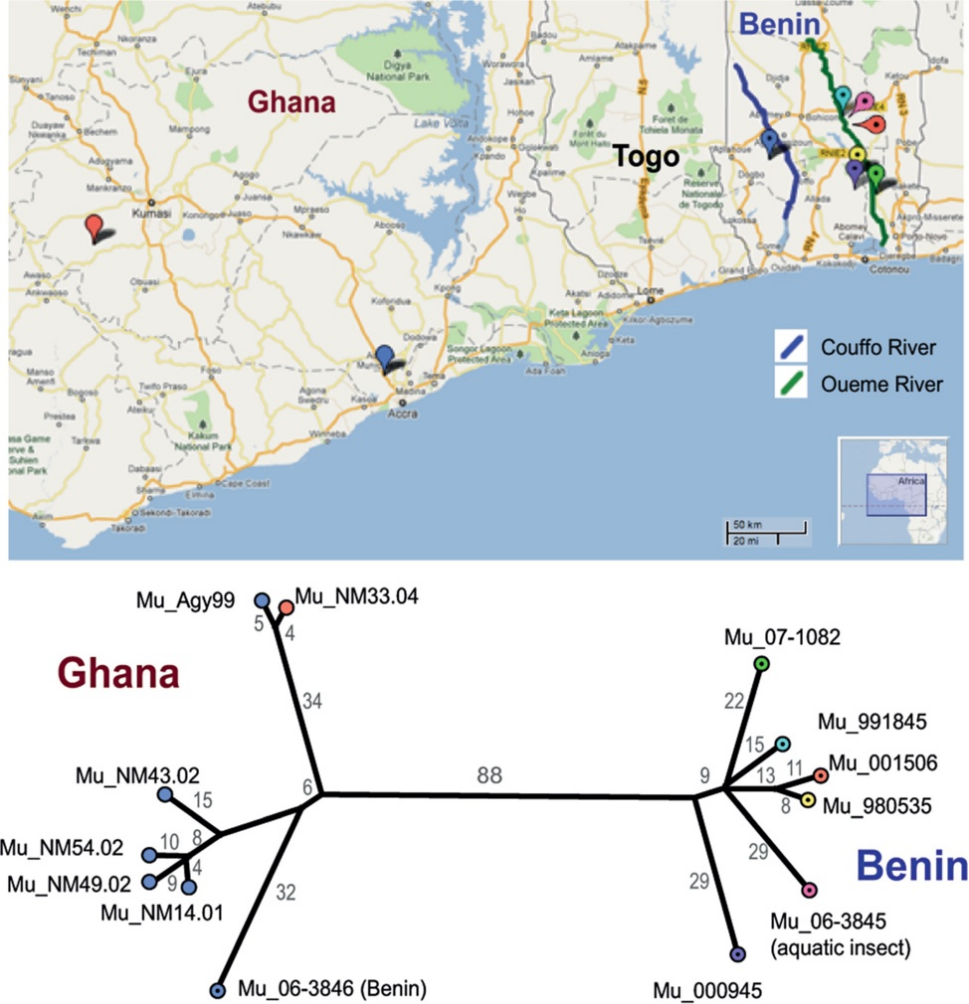
**African isolates tree and map.** Unrooted neighbour joining tree produced by SplitsTree4 [41] using uncorrected ‘P’ distances and based on 396 variable common nucleotide positions from a core of 5,190,553 bp among the 13 isolates from Ghana and Benin. The map shows their geographic distribution with pin colours corresponding to the tree. The isolate Mu_06-3846 is from Benin but clusters with the Ghanian isolates (see text). Map produced using Google Maps.

Through our core genome SNP comparisons we were able to compare isolates within localised geographic regions. Using the same read-mapping approach as described above we defined a 5,190,533 bp core genome and phylogeny for the subset of 13 isolates from Benin and Ghana (Figure 5). Only 396 variable nucleotide positions were identified, with an average pairwise distance of 160 SNPs differentiating isolates between Ghana and Benin. However, Mu_06-3846 isolated in the Couffo valley in Benin, clustered with the isolates from Ghana. The other six isolates from Benin originated from the Zou/Ouémé valley. A previous study has demonstrated a genetic difference between *M. ulcerans* from these two river basins [28]. An explanation for the apparent clustering of the Couffo strain (Mu_06-3846) with the Ghanaian strains could be obtained by sequencing more isolates from the Couffo valley, from Togo (*i.e.* the area between the Couffo valley in Benin and Ghana) and Ghana. The population structure revealed in Figure 5 indicates localized clonal expansion in Ghana and Benin, suggesting that most BU infections in these areas result from local transmission of a single circulating clone, with only occasional transfer of clones between geographic areas. The environmental isolate *M. ulcerans* Mu_06-3845, obtained from an aquatic insect in Benin, clustered with the BU Zou/Ouémé Benin isolates but is distinct from a human isolate obtained from the same region at the same time (Mu_001506). Among this limited set of isolates from the Zou/Ouémé valley in Benin, no finer-scale geographical clustering was observed. This is consistent with a previous study of isolates from an area of comparable size in Ghana [30].

We used the same approach to construct a phylogenetic tree for the mycolactone plasmid pMUM001. No pMUM001 sequences were detected among the four *M. marinum* isolates or *M. ulcerans* isolate Mu_8765 (Lineage 2), indicating that the plasmid was absent from these isolates. Mu_8765 has previously been reported to have lost its plasmid during laboratory passage [26]. All other MPM isolates carried the plasmid, confirming the central role of pMUM001 in the evolution of this complex. A total of 315 pMUM001 SNPs were identified among the MPM isolates, permitting construction of a distance-based phylogenetic tree for the plasmid (Figure 4, inset). The plasmid phylogenetic tree closely matches both the topology and relative branch lengths of the core chromosome tree, consistent with co-evolution of the plasmid and chromosome in each lineage [21]. By comparing read depths for plasmid and chromosome (see Methods), we estimated an average pMUM copy number of 1.8 copies per cell (range 1.3 for Mu_CC240299 to 3.1 for Mu_JKD8071).

### Inferred genomic characteristics of *M. ulcerans* MRCA

First, we identified ancestral SNPs that differentiate the most recent common ancestor (MRCA) of *M. ulcerans* from the rest of *M. marinum,* which may have played a role in evolution of the distinctive phenotypic characteristics of *M. ulcerans*. Using *M. marinum* “M” as a reference, we identified 4,170 such SNPs (including small indels), spanning the entire chromosome and comprising 607 intergenic, 2,254 synonymous, 1,301 non-synonymous and eight SNPs occurring within pseudogenes (Figure 1, Additional file 3: Table S2). This list of mutations, which includes the recently reported hspR_2 regulatory mutation [42], will be important for research exploring functional characteristics of *M. ulcerans*. The identification of these SNPs is also the foundation for evaluation of evolutionary forces acting on specific genes (see later section).

**Figure 6:**
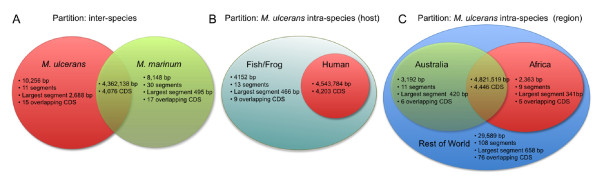
**Venn diagrams, showing DNA shared and unique among isolates.** Venn diagrams showing the unique genomic material in the isolates when partitioned by species, host and region. All regions were identified by finding the core genome of the isolates within the set and removing the pan-genome of all isolates excluded from the set. The unique material for each partition is shown as total size in base pairs, number of segments in partition with a minimum size of 200 bp, the length of the largest segment and the number of CDS with an overlap of >10%. The intersection of the sets was determined by finding the core genome of the union of the sets of isolates and shows the size of the core and the number of CDS with an overlap of >10%. (**A**) Shows the partition between all 30 *M. ulcerans* isolates and all four *M. marinum* isolates. (**B**) Shows the partition between the four *M. ulcerans* fish and frog host isolates and the *M. ulcerans* human host isolates. The human host isolates are a strict subset of the fish and frog isolates with no unique DNA. (**C**) Shows the partitioning between the 10 Australian and 13 African *M. ulcerans* isolates and their separation from the isolates from the rest of the world (6 isolates). The combined African and Australian isolates have no unique DNA when compared to the rest of the world isolates.

Next, we investigated chromosomal regions that were conserved within *M. ulcerans* but absent from *M. marinum*. We identified 11 DNA segments of up to 2,688 bp in length, overlapping 15 CDS (total 10,256 bp) (Figure 6A). The locations of these CDS on the *M. ulcerans* chromosome are shown in Figure 1 and their annotations are described in Additional file 4: Table S3. These *M. ulcerans-*specific chromosomal genes include seven prophage genes from phiMU02, a phage-like polymerase (MUL_0027), a putative lipase (MUL_2832) and six hypothetical proteins. Hence other than the pMUM plasmid, ISs and phiMU02 as already described, *M. ulcerans* isolates have few other species-specific DNA elements and is instead characterised by gene loss. However the eight identified *M. ulcerans*-specific genes may be important for the ecology of *M. ulcerans* and warrant further investigation.

We also explored differences in gene content between *M. ulcerans* isolated from different geographic locations or from different hosts (Figure 6B, 6C, Additional file 5: Table S4). The core genome of *M. ulcerans* isolates known to infect humans comprised a strict subset of the core genome of fish and frog isolates. Nine CDS were found only in the fish/frog isolates and may be assumed to be superfluous for *M. ulcerans* infections in humans, although care needs to be exercised as there were only five fish and frog isolates on which to base this analysis. The products of the nine CDS include five hypothetical proteins, three putative hydrogenases and a putative oxononanoate synthase (BioF2_5). A total of 76 CDS were conserved in *M. ulcerans* lineages 1 and 2 but absent from lineage 3 (which contains only African and Australian BU isolates) (Figure 6C). These data suggest that the African and Australian BU isolates, which represent the majority of the global burden of BU infection in humans, have undergone further reductive evolution. This may be associated with additional adaptation to a more specialised niche, or genetic drift associated with passing through an evolutionary bottleneck. The 76 genes lost (Additional file 5: Table S4) include some involved in metabolic and respiratory processes, which may be no longer required in the more restricted environment occupied by lineage 3 isolates [43]. We also investigated novel *M. ulcerans* genes not present in the reference genome (Agy99, an African BU isolate from Lineage 3) via annotation and comparison of *de novo* assemblies of the novel isolates. The African and Australian isolates, also from Lineage 3, had no novel CDS apart from a putative integrase in six of the ten Australian isolates. The remaining *M. ulcerans* isolates from other lineages had many more novel CDS as follows; Mu_JKD8071 (128), Mu_06-3844 (93), Mu_CC240299 (93), Mu_L15 (13), Mu_1G897 (8), Mu_8765 (3). The numbers of novel CDS largely reflects the isolates’ similarity to the reference genomes as shown in Additional file 2: Figure S2.

Figure 6 also shows the largest segments of DNA that could be used to discriminate between isolates by species, host and region (genomic locations are listed in Additional file 5: Table S4). For example, to discriminate between a *M. ulcerans* and a *M. marinum* isolate, there is a 2,688 bp segment that is present in all *M. ulcerans* isolates and absent from all other *M. marinum* isolates. Together with previously described deletions, these DNA segments could provide the basis of a set of DNA diagnostic tests to identify the species, host or region of an unknown isolate.

**Figure 7:**
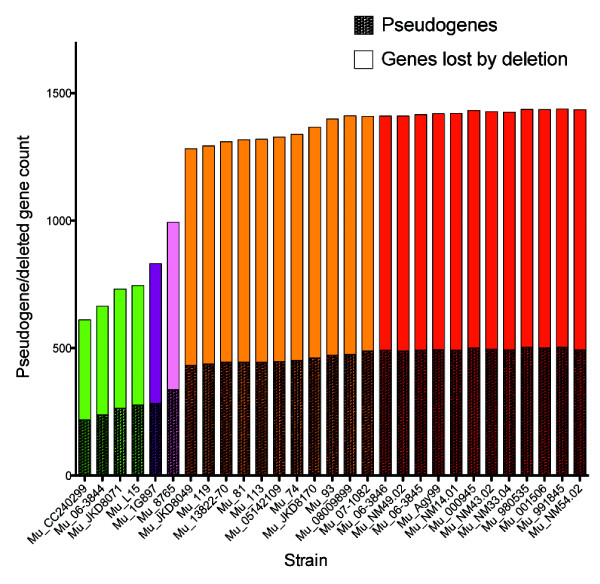
**Inferred pseudogenes and deleted genes.** Counts of inferred pseudogenes and deleted genes among the study isolates. The counts were inferred by analysis of SNPs that would render *M. marinum* reference CDS inactive and read coverage mapping showing partially or fully deleted CDS. The isolates are coloured by groups; African isolates – red, Australian isolates – gold, French Guiana isolate – purple, Japanese isolate – pink, fish and frog isolates – green.

The *M. ulcerans* Agy99 reference contains the second highest percentage (13.8%) of pseudogenes within a diverse set of 64 prokaryotic genomes [44]. *M. leprae* has the highest percentage (36.5%) and represents an extreme case of genome reduction combined with loss of function. The pseudogenes present within the MRCA of the *M. ulcerans* isolates provide insights into the loss of function that may have been instrumental in the adaptation of *M. ulcerans* from the more generalist *M. marinum*. MRCA pseudogenes were inferred by assuming the inactivation of *M. marinum* CDS (intact in all *M. marinum* strains) via mutations in the *M. ulcerans* isolates affecting start codons, creating frame-shifts or introducing premature stop codons. This analysis resulted in a list of 185 putative ancestral pseudogenes and/or deletions that were identified in all *M. ulcerans*, of which 83 were absent in Agy99 due to gene deletion (Additional file 6: Table S5). Hence at least 25% of the pseudogenes present in *M. ulcerans* Agy99 - or lost from this isolate by deletion - are shared by all extant *M. ulcerans* isolates and indicates that significant remodelling and adaptation through reductive evolution was occurring in the *M. ulcerans* MRCA before the global radiation of the species. The number of inferred pseudogenes and deleted genes per isolate is shown in Figure 7 and shows that lineage 3 strains have all lost substantially more coding potential than lineage 1 strains. An examination of the distribution of these ancestral pseudogenes by functional group shows that CDS belonging to one specific group, “cell wall and cell wall processes”, are significantly overrepresented (35%, compared with ~22% for all CDS in *M. marinum* M and *M. ulcerans* Agy99). Table 2 summarizes some of the key CDS losses that may have played a role in the early adaptive responses of *M. ulcerans* following the acquisition of pMUM and a pathway of reductive evolution and includes loss of CDS required for synthesis of selenocysteine-containing proteins, anaerobic respiration, stress responses, intracellular survival, acylation of lipoproteins and biosynthesis of isoprenoids.

**Table 2:** **Noteworthy genes or loci absent in the *M. ulcerans* MRCA**

Gene(s)	Locus_tag(s)	Comments	Reference
*selA*	MMAR_5190	selenocysteine synthase, required for the synthesis of proteins containing selenocysteine.	[1]
	MMAR_5194 MMAR_5195	Formate dehydrogenase alpha and beta subunits, selenocysteine-containing. Likely role in anaerobic growth.	[1]
	MMAR_2615	Anaerobic dehydrogenase (putative nitrate reductase), possible role in anaerobic growth.	
*lgt*	MMAR_2416	Lgt acylates prolipoproteins, thus key role in lipoprotein synthesis. Lgt mutant of *S. aureus* is less immunogenic.	[45-47]
*plcB_2,*	MMAR_1485	*M. marinum* has six plc genes. Four of these are absent in all *M. ulcerans*. Phospholipase C enzymes can cause direct or indirect enzymatic hydrolysis of host cell membrane phospholipids and appear important for mycobacterial intracellular survival.	[48]
*plcB_5,*	MMAR_3656		
*plcB_6,*	MMAR_4722		
*plcB_3*	MMAR_0284		
*cstA*	MMAR_1616	Carbon starvation protein, CstA. In *E. coli*, *cstA* encodes a peptide transporter and is induced by carbon starvation. Maybe part of a redundant stress response system in *M. ulcerans.*	[49]
*cueO*	MMAR_1618	Multicopper oxidases protect against oxidative stress. This enzyme has functions in tolerance to copper, and iron and manganese oxidation in a range of bacteria. It catalyses the oxidation of cuprous copper, ferrous iron and diphenolic compounds. In Salmonella, a *cueO* deletion mutant is less virulent in mouse model. Divergent transcriptional arrangement with *cstA*.	[50]
*idsB1,*	MMAR_3212	Catalyzes the trans-addition of three molecules of IPP onto DMAPP to form geranylgeranyl pyrophosphate which is a precursor of the ether-linked lipids. Impact here of diverting all isoprenoid biosynthesis to the non-mevalonate pathway is not known. Possibly more favourable energetically if spending cellular resources on mycolactone synthesis.	[1]
*idsB2,*	MAR_3219		
*idsA1*	MMAR_5095		

### Impact of IS*2404* and IS*2606* on *M. ulcerans* genome architecture

**Figure 8:**
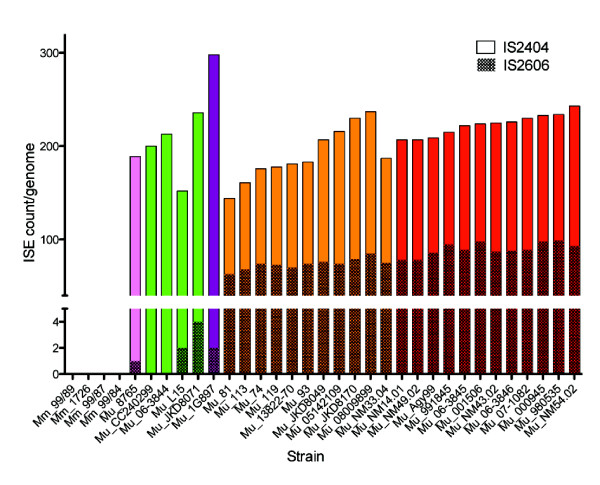
**IS *2404* and IS *2606* distribution among isolates.** Counts of IS*2404* and IS*2606* among the study isolates. The counts were inferred from coverage of reads mapping to each IS. The isolates are coloured by groups; African isolates – red, Australian isolates – gold, French Guiana isolate – purple, Japanese isolate – pink, fish and frog isolates – green. IS*2404* and IS*2606* were not detected in the non-MPM *M. marinum* isolates

A key differentiator between *M. marinum* and *M. ulcerans* is the presence of multiple copies of IS*2404* and IS*2606* in *M. ulcerans* leading to genome plasticity and remodelling. We estimated the copy number of these IS in each isolate and confirmed that neither IS was present in any of the *M. marinum* isolates, as reported previously [1]. We found that *M. ulcerans* genomes from Lineages 1 and 2 had only 1–4 copies of IS*2606*, while a massive expansion has occurred in Lineage 3 resulting in 63–98 copies per genome (Figure 8). Conversely, we identified a large number (41–81) of novel IS*2404* insertions in Lineage 1 and 2 isolates (relative to the Agy99 reference) but only a single novel IS*2404* insertion in 9 out of the 23 Lineage 3 isolates. These data confirm that ISs are continuing to modify the *M. ulcerans* genome. The sequence data of lineage 1 isolates Mu_CC240299 and Mu_06-3844 each contain a single copy of IS*2606* that could be located within the pMUM plasmid but no copies were found within the chromosome (see Methods). As well as containing IS*2404*, the pMUM plasmid contains between one and eight copies of IS*2606* and is therefore the likely origin of IS*2404* and IS*2606* in *M. ulcerans* chromosomes.

### *M. ulcerans* genes under selective pressure

Identification of CDS that are under selective pressure can give additional insights into the specific nature of the environment(s) an organism encounters. The ratio dN/dS is the number of non-synonymous substitutions per non-synonymous site (dN) to the number of synonymous substitutions per synonymous site (dS); hence dN/dS > 1 implies diversifying selection, while dN/dS < 1 implies purifying selection through loss of mutations that cause changes at the protein level. To compare selection in the *M. marinum* isolates against the *M. ulcerans* isolates, two likelihood function (LF) models were generated that compared a constant dN/dS across all isolates against a model where dN/dS differed across the tree. The dN/dS value across a core set of 2,379 CDS for the full tree was 0.181 (constant model) while the values for the differing sub-branches (varying model) were 0.093 for *M. marinum* isolates, 0.484 for African Lineage 3 *M. ulcerans* isolates, 0.515 for Australian Lineage 3 *M. ulcerans* isolates and 0.459 for *M. ulcerans* lineages 1–2. This is consistent with relaxation of purifying selection in *M. ulcerans* compared to *M. marinum* (log likelihood ratio statistic = 8988.6, degrees of freedom = 63, *p* < 1e-6), however the younger age of *M. ulcerans* could also explain this result [51]. This dN/dS variation was consistent across all annotated functional groups: (*M. ulcerans* isolates dN/dS, *M. marinum* isolates dN/dS) conserved hypotheticals (0.538, 0.191), cell wall and cell processes (0.524, 0.165), lipid metabolism (0.484, 0.160), regulatory proteins (0.470, 0.160), intermediate metabolism (0.469, 0.148), information pathways (0.402, 0.128). Next we compared dN/dS at the level of individual CDS. Our model parameter start values were taken from the LF and the tree previously used to compare regions using the full set of CDS. This improved the ability of the new LF to be more rapidly and reliably optimised. Of the 2,379 CDS examined, 172 CDS had a dN/dS > 1 for the *M. ulcerans* isolates (Additional file 7: Table S6). This set was dominated by two functional groups, ‘cell wall and cell processes’ (44 CDS) and ‘conserved hypotheticals’ (56 CDS). Notable CDS in this set of genes under diversifying selection included *esxE* and *esxF* that encode WXG100 domains and are orthologs of the secreted T-cell antigens EsxA and EsxB. The stress response sigma factor *sigM* was also identified as under diversifying selection, perhaps indicating remodelling of the SigM regulon in *M. ulcerans*[2] (Figure 1). In *M. tuberculosis, sigM* appears to positively regulate *esxE* and *esxF* expression while negatively regulating certain non-ribosomal peptide synthetases and polyketide synthase genes involved in biosynthesis of cell wall lipids [52,53]. Lipoproteins, another class of cell wall-associated molecule, also featured among the genes displaying evidence of diversifying selection (*lppN, lppD, lprA*) and perhaps links to the predicted loss of Lgt activity and the ability to acylate prolipoproteins (Table 2).

We also examined CDS that contained only non-synonymous SNPs, prohibiting calculation of a dN/dS ratio, or were not core to *M. ulcerans*, and compiled a list of additional CDS with an overabundance of non-synonymous SNPs, sorted by the difference between non-synonymous and synonymous SNPs (see Methods). The 50 most variable proteins mirrored the dominant functional groupings of the high dN/dS analysis (Additional file 7: Table S6), including *esxE/F* as well as enoyl-CoA hydratase *echA4_2* and *ssp*. Ssp (signal peptide protease IV) is a serine protease required for digestion of cleaved signal peptides, indicating that *M. ulcerans* may be under pressure to modify their secretome. Also of interest is *mymT*, encoding a copper-binding protein, a cytoplasmic metallothionein and in *M. tuberculosis*, sequesters excess copper in the bacterial cell [54].

The close relationship between *M. tuberculosis* and the MuMC led us to investigate whether the orthologous human T cell epitopes for *M. tuberculosis* were conserved in *M. ulcerans*. The genes encoding antigens in human pathogens tend to be under diversifying selection in order to evade host immunity and it has been suggested that this is not the case with *M. tuberculosis*[55]. We found 20 MuMC orthologs (>80% AA identity) of the 86 human T-cell antigens in *M. tuberculosis* H37Rv (Additional file 8: Table S7). These orthologs all had very low dN/dS ratios (*M. marinum* isolates median 0.05, range 0.15, *M. ulcerans* isolates median 0.17, range 0.95) suggesting that these CDS are under purifying selection in the MuMC as they are in *M tuberculosis*.

## Discussion

Whole genome sequencing and comparison of this large collection of isolates has shown that all mycolactone-producing mycobacteria (collectively referred to in this study as *M. ulcerans*) evolved from a common *M. marinum* progenitor by a combination of horizontal gene transfer (pMUM and phage), IS-mediated deletion and point mutation (Figure 4). These evolutionary processes resulted in extensive gene loss (185 CDS), changes in gene expression (*e.g. hsp18* and possibly *sigM*) and likely changes in gene function through positive selection, establishing *M. ulcerans* as a highly specialized, niche-adapted mycobacterium. Our data indicate these changes were followed by global dispersal of *M. ulcerans* and further diversification and adaptive evolution.

Scrutiny of the types of genes gained, lost or modified in conjunction with other experimental evidence provides some clues regarding the nature of the niche environment in which the *M. ulcerans* MRCA was able to flourish and in which today’s isolates still survive. Foremost among the DNA gained was in the pMUM plasmid. The acquisition of this plasmid occurred early in the evolution of *M. ulcerans* as demonstrated by the congruent tree topologies inferred from chromosome and plasmid sequence alignments (Figure 4). The synthesis of mycolactone, a potent immunosuppressive small molecule encoded in the pMUM plasmid, presumably gave a population of *M. marinum* cells the ability to persist in a place their generalist relatives could not. In previous research we have described how recombination and gene conversion has shaped the unusually repetitive gene structure of the mycolactone PKS, such that the 110 kb, three-gene PKS locus on pMUM comprises only 10 kb of unique sequence. This unusual gene structure and its resultant instability are strongly suggestive of intense selection acting on *M. ulcerans* populations to maintain mycolactone production [5,56,57].

The loss of the mevalonate pathway for synthesis of isoprenoid lipids was originally observed in the genome of *M. ulcerans* Agy99 [1]. In the current study we show that this trait was established in the *M. ulcerans* MRCA and was probably a key adaptive response of the bacterium following the acquisition of pMUM (Table 2). It is possible that loss of this metabolic capacity freed essential resources for critical mycolactone synthesis or alternatively that these pathways and metabolites were redundant in the niche environment occupied by *M. ulcerans*. Other potentially significant gene losses in *M. ulcerans* include selenocysteine synthase (*selA*), required for the synthesis of proteins containing selenocysteine, and the linked genes encoding the alpha and beta subunits of a putative selenocysteine-containing formate dehydrogenase, with a possible role in anaerobic growth; mutations that suggest the *M. ulcerans* MRCA lost the ability to grow anaerobically.

It is also striking that genes associated with the intracellular lifestyle of mycobacterial pathogens such as *M. marinum* and *M. tuberculosis* have been lost in *M. ulcerans*. Genes predicted to be inactive include four phospholipase enzymes (PlcB_2,3,5&6) and *cueO* (Table 2). As well as playing roles in intracellular replication in other bacteria, these CDS are also components of the bacterial cell wall. There appears to have been significant selective pressure on *M. ulcerans* to reduce or change its cell wall and cell surface antigenic profile. In this respect another noteworthy pseudogene conserved in *M. ulcerans* is *lgt* (MUL_1594). Lgt is a prolipoprotein diacylglyceryl transferase that acylates prolipoproteins at a conserved N-terminal cysteine [45]. An *lgt* mutation in *Staphylococcus aureus* causes growth rate attenuation, an accumulation of prolipoproteins in the culture supernatant, and reduced activation of innate immune responses [46,58]. The loss of *lgt* in *M. ulcerans* might therefore lead to aberrantly or non-acylated lipoproteins with reduced immunogenicity, like the *S. aureus* mutant.

The modification of the cell wall appears to have continued in the BU-associated *M. ulcerans* lineage 3 genomes, which contain an additional 589 pseudogenes or deleted regions of which 30% are predicted to have encoded antigens or cell wall associated proteins, including EsxA_2, EsxA_3, and Hspx_1. The deep branching lineage and clonal nature of the African and Australian lineage 3 isolates, which are most commonly involved in human infections, have the signature of passing through a second evolutionary bottleneck: gene deletions, further loss of gene function, chromosomal rearrangements and the expansion of another IS (IS*2606* from the pMUM plasmid). Each of the *M. ulcerans* lineages probably represents different ecotypes, reflecting adaptation to related but distinct niche environments. It may be that each lineage is best described as an *M. ulcerans* ecovar.

We tried to identify a temporal signal in our sequence data to estimate divergence dates for particular lineages of the MuMC. However, while our phylogenetic inferences were highly robust (100% bootstrap values for major branches of our tree, Figure 4) no linear correlation between branch length and year of isolation was observed. We suspect there is variation in the effective number of generations per year across the complex, perhaps related to different niches, reservoirs or modes of living. Such variation has recently been observed in *M. tuberculosis*[59]. The lack of a temporal signal in these data raises doubts around previous estimates of divergence time that have assumed a constant molecular clock rate among the lineages of the complex [26,34].

However, sequence comparisons did reveal a compelling correlation between genes undergoing positive selection - as revealed by dN/dS analysis - and those CDS inactivated or deleted (Additional file 6: Table S5, Additional file 7: Table S6), with mutations in all these groups skewed towards CDS involved in cell wall and lipid biosynthesis. These patterns point to significant selective pressures acting on *M. ulcerans* populations to devote resources (substrate and energy) towards the synthesis of mycolactones and modification of cell wall structures. Intriguingly, many of the cell wall metabolites lost via mutation are known to be highly antigenic in other mycobacteria. One interpretation of these observations is that the bacteria are responding to pressures from a host immune system, a point argued in a previous study [60]. Furthermore, when one considers that mycolactone is a potent immune suppressor with reported specificity for a mammalian microRNA that controls T-cell chemotaxis [61], this in turn leads to the idea that the niche occupied by *M. ulcerans* is a higher organism with a complex immune system. The discovery that Australian possums inhabiting BU endemic areas are susceptible to BU disease and harbour large number of *M. ulcerans* in their gastrointestinal tracts is consistent with this idea [14]. Nevertheless, these arguments are not consistent with the significant lack of variation seen among *M. ulcerans* proteins with putative T-cell epitopes (Additional file 8: Table S7), where an immune escape hypothesis would predict hypervariability not hyperconservation in these regions.

This study has also reinforced the close relationship between isolate origin and genotype for *M. ulcerans* strains that cause Buruli ulcer, notably those from Africa and Australia, where multiple isolates from one region were sequenced. The complete resolution of strain differences afforded by whole genome sequencing has shown how the genotype of *M. ulcerans* strains from two African countries correlate with place of origin. Isolates from the east of Benin are distinct from isolates in the West of the country or from a different country (Ghana, Figure 5). These findings suggest - as also demonstrated in a previous study in Ghana - that *M. ulcerans* transmission and microevolution generally occurs at a local level and therefore the source of the bacterium is somewhat fixed within a local region [30]. This observation should guide our thinking regarding the source of the bacteria in BU endemic areas, indicating that animal reservoirs of *M. ulcerans* are unlikely to be highly mobile. Yet, one should also consider that the relative paucity of genomic differences between isolates from Ghana and Benin also reflects the relatively recent spread of the bacteria across this entire region. Efforts to establish the rate of mutation of these isolates or genome analysis of a more temporally and spatially diverse collection of isolates from this region might help estimate the amount of time *M. ulcerans* has been extant in West Africa.

## Conclusions

This study has given us our most comprehensive insight yet into the *M. ulcerans*-*M. marinum* complex. The isolates examined in the study covered a wide geographical diversity, and while future studies should include *M. ulcerans* isolates from other endemic countries together with more *M. marinum* isolates, whole genome comparisons of 35 isolates across the genetic diversity of the complex have yielded nucleotide-level granular detail of each isolate and their relatedness to each other. We have shown that all MPM are a single lineage whose divergence from *M. marinum* was characterised by acquisition of the pMUM plasmid, which conferred the ability to synthesise mycolactones (largely responsible for the immunomodulatory properties of *M. ulcerans*) and also introduced IS*2404* and IS*2606* into the chromosome; this was then followed by IS*2404* and IS*2606* expansion and extensive loss and modification of gene function, consistent with an evolutionary bottleneck and adaptation to a new niche. Based on these shared features we propose that all members of this lineage should be considered *M. ulcerans*. Furthermore, we suggest that *M. ulcerans* sublineages (such as the three we have identified in this study) should be considered *M. ulcerans* ecovars.

Examination of the classes of genes lost and modified in the *M. ulcerans* MRCA suggests a bacterial population occupying a niche environment that is aerobic, osmotically stable, dark (at least for lineage 3 isolates as shown by loss of UV-protecting pigment genes in a previous study) and possibly extracellular given the number of genes known to be involved in intracellular survival lost from *M. ulcerans*[1,13,21]. Support for an extracellular niche is also found in two separate studies showing *M. ulcerans* elaborates a mycolactone-rich extracellular matrix and specifically expresses a surface protein that promotes adherence during initial stages of biofilm formation [42,62]. Furthermore, it is difficult to ignore the striking depletion of immunogens and modification of cell-wall proteins in *M. ulcerans* that may be subject to interactions with the immune defences of a host organism.

This study has clarified our understanding of the origins of an emerging human pathogen, provided important insights into the search for the reservoir(s) of this pathogen and generated a significant resource for future research. It is clear that *M. ulcerans* is not a generalist, saprophytic mycobacterium but a highly specialized occupant of a protected niche. This concept should be considered in ongoing research efforts to pinpoint the reservoir(s) of *M. ulcerans* and to understand the transmission of BU.

## Methods

Several bioinformatics approaches were used to analyse the sequence data. The workflow we developed is summarised in Additional file 9: Figure S1 and involved analysis of sequence differences and similarities between all strains. A mapping-to-reference approach was applied as well as *de novo* assembly of the sequenced reads. A summary of the isolate data is presented in Additional file 1: Table S1, including the number of reads and the *de novo* assembly statistics for each strain.

### Bacterial isolates and sequencing

The isolates of *M. ulcerans* and *M. marinum* used in this study are described in Table 1. Genomic DNA was prepared from at least 50 mg (wet weight) cell pellets, harvested from mycobacteria that were cultivated on egg-yolk agar media as previously described [42].

Genome sequencing of the isolates (except for Mu_DL045) was performed using an Illumina GAIIx DNA sequencer with 36, 76, or 100 cycle paired-end chemistry, generating read lengths of 35–101 nucleotides and 16-240x mean read depth per isolate. Sequencing statistics are provided in Additional file 1: Table S1. Mu_DL045 was sequenced using the Ion Torrent platform to generate single-ended reads. Read data for the study isolates (Mu_06-3845, Mu_06-3846, Mu_07-1082, Mu_113, Mu_119, Mu_74, Mu_81, Mu_93, Mu_05142109, Mu_JKD8170, Mu_08009899, Mu_JKD8049) have been deposited in the NCBI Sequence Read Archive (SRA) under accession SRP004497 [63].

Prior to further analysis, reads were filtered to remove those containing ambiguous base calls, any reads <24 nucleotides in length and reads containing only homopolymers. All reads were trimmed from the 3’ end removing bases with the Illumina Read Segment Quality Control Indicator “B”.

### Read mapping

An in-house Python utility called Nesoni, which uses SHRiMP2 [64] for read mapping, was used to map novel sequence reads to reference genomes [65]. Nesoni identified SNPs and indels up to ~10 bp and predicted the consequences of SNPs and indels on CDS, e.g. frameshifts, premature stop codons and non-synonymous codon changes. Five reference genomes and fragments were used for read mapping, (sequence name, Genbank accession number) (*M. ulcerans* Agy99, CP000325), (*M. marinum* strain “M”, CP000854), (*pMUM001* plasmid, BX649209), (*pMUM002* plasmid, EU271968) and (*pMUM003* plasmid, EU271967). To facilitate comparison of *M. marinum* and *M. ulcerans* reference genomes using Nesoni, short read data for *M. marinum* “M” was simulated via *in silico* shredding of the reference genome to generate a set of 100 bp short reads (read depth x15 insert length 280 bp) using a utility function available within Nesoni. Average read depth and the number of SNPs detected by mapping to *M. ulcerans* Agy99 and *M. marinum* M are given in Additional file 1: Table S1.

The number of reads mapping to unique regions of the plasmids was used to infer plasmid copy number. The ratio of the mode of read depth for the reference plasmid (pMUM002 for Mu_06-3844, Mu_CC240299, Mu_JKD8071, Mu_L15, Mu_1G897 and pMUM001 for all other *M. ulcerans* strains) was compared to the mode of read depth for the chromosome.

### SNP analysis

Variant sites (SNPs and indels) identified using Nesoni were concatenated to form a multiple alignment, from which a phylogenetic tree was constructed using SplitsTree4 (neighbour-joining tree based on uncorrected P distances) [66]. All major bifurcations had >95% bootstrap support based on 1,000 runs. The tree shown in Figure 4 is based on SNPs identified using *M. marinum* M as the reference, however the same tree was recovered when using *M. ulcerans* Agy99 as the reference. SNPs were classified by their position on the annotated references of *M. ulcerans* Agy99 and *M. marinum* “M” and identified as ‘intergenic’, ‘coding’ or ‘pseudogene’; ‘coding’ SNPs were further divided into synonymous, non-synonymous or nonsense SNPs. All SNPs called within annotated ISs or PE/PPE genes were excluded from SNP analysis due to the unreliability of read mapping in repetitive regions [8,67].

### *De novo* assembly of short-read sequences

Genomes were assembled using Velvet [68] with the help of VelvetOptimiser [69] to select appropriate parameter values for k-mer size, expected coverage and coverage cutoff. The Velvet assemblies yielded an average N50 of 18,399 bp; assembly statistics are given in Additional file 1: Table S1. Repetition of hundreds of copies of IS*2404* and IS*2606* within the *M. ulcerans* isolates was problematic and restricted the contig lengths, as the size of sequenced DNA fragments (≤240 bp) was much smaller than the length of the ISs (1365–1444 bp). To identify IS disrupted contigs, paired reads were mapped to consensus IS*2404* and IS*2606* sequences. Where a read was mapped to the IS but its partner read was not, the unmapped partner reads were pooled and *de novo* assembled to identify the DNA sequences that flank the IS sequences. This analysis demonstrated that 51% (11% s.d.) of the contig breakpoints terminated at either IS*2404* or IS*2606*. Mycobacterial genomes contain hundreds of PE/PPE genes, which pose problems for sequencing, read mapping and assembly due to their repetitive, low G + C content 3’ sequences [70]. Hence PE/PPE genes were excluded from most downstream analysis including SNP calling and gene content analysis. Contigs were annotated by Prokka, an in-house bacterial genome annotation pipeline using Glimmer, EasyGene, Orpheus and GeneMark.

### Core and pan genome analysis

The core genome was defined as all positions in the reference genomes that were covered by ≥3 reads in all isolates; this information was derived from depth plots generated by Nesoni. The set of genomic material conserved in one set of isolates, A, but absent from a second disjoint set of isolates, B, was obtained by taking the core genome of A minus (all positions conserved among A) and removing any bases present in B.

To define the pan genome, CDS annotated in the reference or *de novo* assembly contigs were subjected to ortholog clustering. CDS were translated to amino acid sequences and clustered using CD-Hit [71] with a 70% amino acid sequence identity threshold. A core set of *M. ulcerans* CDS was defined by identifying all clusters containing a *M. ulcerans* reference CDS and one or more CDS fragments, with a combined length within 15% of the reference sequence, from each novel *M. ulcerans* genome.

To assess the completeness of the set of study isolates, we calculated the median number of new genes added to the pan-genome for every additional genome, using 1,000 permutations of the study isolates (*M. ulcerans* and *M. marinum*). A trend was modelled by fitting a power curve to estimate the exponent, indicating whether the pan genome is ‘open’ or ‘closed’ [37,38].

### dN/dS analysis

The relative rates of change at synonymous and non-synonymous sites (dN/dS) within protein coding regions was calculated using the BioPerl module Bio::Align::DNAStatistics and the PyCogent toolkit [72]. The dN/dS analysis was performed for both individual CDS (using BioPerl and PyCogent) and for aggregate concatenated CDS (using PyCogent). To test for selection across multiple isolates using multiple CDS, analysis was restricted to an alignment of DNA sequences from all CDS that were present, without a premature stop codon, in all isolates (2,379 CDS). This alignment, together with an unweighted neighbour-joining tree of the isolates, was provided as input to PyCogent modules that calculated the dN/dS values across subsets of the isolates. The CNF codon substitution model was used for all analyses [72]. A representative set of study isolates was used: *M. marinum* Mm_1726, Mm_99/84, Mm_99/87, Mm_99/89; *M. ulcerans* lineage 1 Mu_CC40299, Mu_JKD8071, Mu_DL045, Mu_1G897; *M. ulcerans* lineage 2 Mu_8765; *M. ulcerans* lineage 3 Mu_05142109, Mu_Agy99. This alignment, together with the corresponding subset of the tree and a codon substitution model, was used to create a likelihood function (LF) which models selection using the PyCogent framework [73]. By specifying how the dN/dS parameter values of the model vary, optimisation of the LF can estimate dN/dS across the whole tree, a sub branch of isolates or individual isolates. The dN/dS metric cannot identify CDS that contained only non-synonymous SNPs but have no synonymous SNPs. As an alternative method of identifying CDS with an overabundance of non-synonymous SNPs, we calculated the average difference between the number of non-synonymous and synonymous SNPs normalised for the length of CDS. Functional groups used to classify CDS were defined by the reference genome annotations and based on COG v1.0 / CDD groupings.

### Pseudogene identification

In order to identify the pseudogenes in the *M. ulcerans* MRCA we examined the CDS SNP changes that were common in all *M. ulcerans* isolates but not present in any *M. marinum* isolates. All PE/PPE, ISs and *M. marinum* pseudogenes were excluded from this analysis. The remaining *M. marinum* CDSs were examined to determine whether the SNPs called by read mapping the *M. ulcerans* isolates onto *M. marinum* resulted in a change to the CDS that would likely render it inactive within the *M. ulcerans* isolates; namely (i) indels that result in frameshifts, (ii) premature stop codons truncating encoded protein, (iii) a change to the start codon, (iv) an IS insertion or (v) a deletion of part or whole of the CDS.

## **Abbreviations**

ANI, Average nucleotide identity; BU, Buruli ulcer; CDS, Protein-coding DNA sequence; Indel, Insertion or deletion in genome; IS, Insertion sequence; LF, Likelihood function; MLST, Multi-locus sequence typing; MPM, Mycolactone producing mycobacteria; MRCA, Most Recent Common Ancestor; MTBC, Mycobacterium tuberculosis complex; MuMC, Mycobacterium ulcerans-Mycobacterium marinum complex; N50, The length of the contig such that 50% of the assembly is contained in contigs of length N50 or greater.

## **Competing interests**

The authors declare that they have no competing interests.

## **Authors' contributions**

KDD co-wrote the manuscript and performed the bioinformatic analysis. TS and wrote some of the analysis tools. KEH carried out the Bayesian analysis and co-wrote the manuscript. JAMF and CJL provided materials and assisted with study design. ME, FP, DY, GP provided materials and co-wrote the manuscript. TPS conceived the study, analysed the data and co-wrote the manuscript. All authors read and approved the final manuscript.

## Supplementary Material

Additional file 1: Table S1 **Strain table and summary statistics.** Isolates used in this study with sequencing summary statistics. Click here for file

Additional file 2: Figure S2 **Percentage DNA difference between isolates.** Scatter Plot showing the percentages of DNA missing from references *M. marinum* “M” (y-axis) and *M. ulcerans* Agy99 and *pMUM001* plasmid (x-axis). The percent missing is calculated by taking the number of zero coverage positions in the short read mapping to reference and dividing by total length of the reference. The dotted lines show the percentage missing from either reference to distinguish the *M. marinum* isolates from the MPM isolates. The clusterings are coloured as follows with the number in each cluster in brackets; *M. marinum* isolates (5) – blue, Fish and frog isolates (5) – green, Japanese isolate (1) – pink, French Guiana isolates – purple, Australian isolates (10) – gold, African Isolates (13) – red. Click here for file

Additional file 3: Table S2 **Core SNPs.** Listing of core SNPs and short indels common to all *M. ulcerans* isolates when mapped to references *M. ulcerans* Agy99 and *M. marinum* M. Click here for file

Additional file 4: Table S3 ** *M. ulcerans* specific CDS and features.** List *M. ulcerans* Agy99 reference annotated features found in all *M. ulcerans* isolates but not found in any of the *M. marinum* isolates. Click here for file

Additional file 5: Table S4 **Diagnostic regions.** List of nucleotide diagnostic regions distinguishing strains between various strain groups: *M. marinum* strains, *M. ulcerans* strains, African strains, Australian strains, strains from other regions, human host strains and fish or frog host strains. Click here for file

Additional file 6: Table S5 **Ancestral Pseudogenes.** List of putative ancestral pseudogenes. Click here for file

Additional file 7: Table S6 **High dN/dS CDS and CDS with high non-synonymous SNPs.** List of core *M. marinum* CDS with dN/dS > 1.0 in representative *M. ulcerans* isolates (Mm_1726, Mm_99/84, Mm_99/87, Mm_99/89, Mu_CC40299, Mu_JKD8071, Mu_DL045, Mu_1G897, Mu_8765, Mu_05142109, Mu_Agy99). Click here for file

Additional file 8: Table S7 ** *M. marinum* orthologs of *M. tuberculosis* T-cell antigens**. List of *M. tuberculosis* T cell antigens with orthologs in *M. marinum* ‘M’ and *M. ulcerans* Agy99. Orthologs have > 80% amino acid identity. Click here for file

Additional file 9: Figure S1 **Work Flow.** Schematic of the workflow with intermediate stages of the data shown in blue boxes. The inputs to the process are shown in red and comprise the annotated reference genomes and the short read sequencing data of the study isolates. The results are shown in green and include the phylogeny of the complex, novel CDS within each of the isolates, core and accessory genomes of the complex and putative ancestral pseudogenes. Click here for file
