# Supplementary figures and images for: On the origin of Mycobacterium ulcerans, the causative agent of Buruli ulcer
Source: BMC Genomics. 2012 Jun 19;13:258. doi: 10.1186/1471-2164-13-258 (PMC3434033; doi:10.1186/1471-2164-13-258)

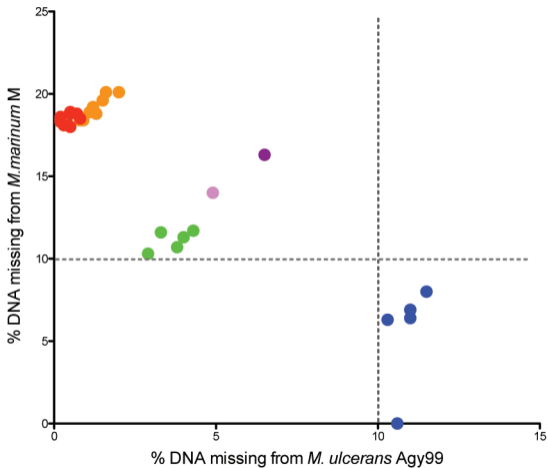

Supplement: Additional file 2: Figure S2 — Percentage DNA difference between isolates. Scatter Plot showing the percentages of DNA missing from references M. marinum “M” (y-axis) and M. ulcerans Agy99 and pMUM001 plasmid (x-axis). The percent missing is calculated by taking the number of zero coverage positions in the short read mapping to reference and dividing by total length of the reference. The dotted lines show the percentage missing from either reference to distinguish the M. marinum isolates from the MPM isolates. The clusterings are coloured as follows with the number in each cluster in brackets; M. marinum isolates (5) – blue, Fish and frog isolates (5) – green, Japanese isolate (1) – pink, French Guiana isolates – purple, Australian isolates (10) – gold, African Isolates (13) – red. [file 1471-2164-13-258-S2.pdf]

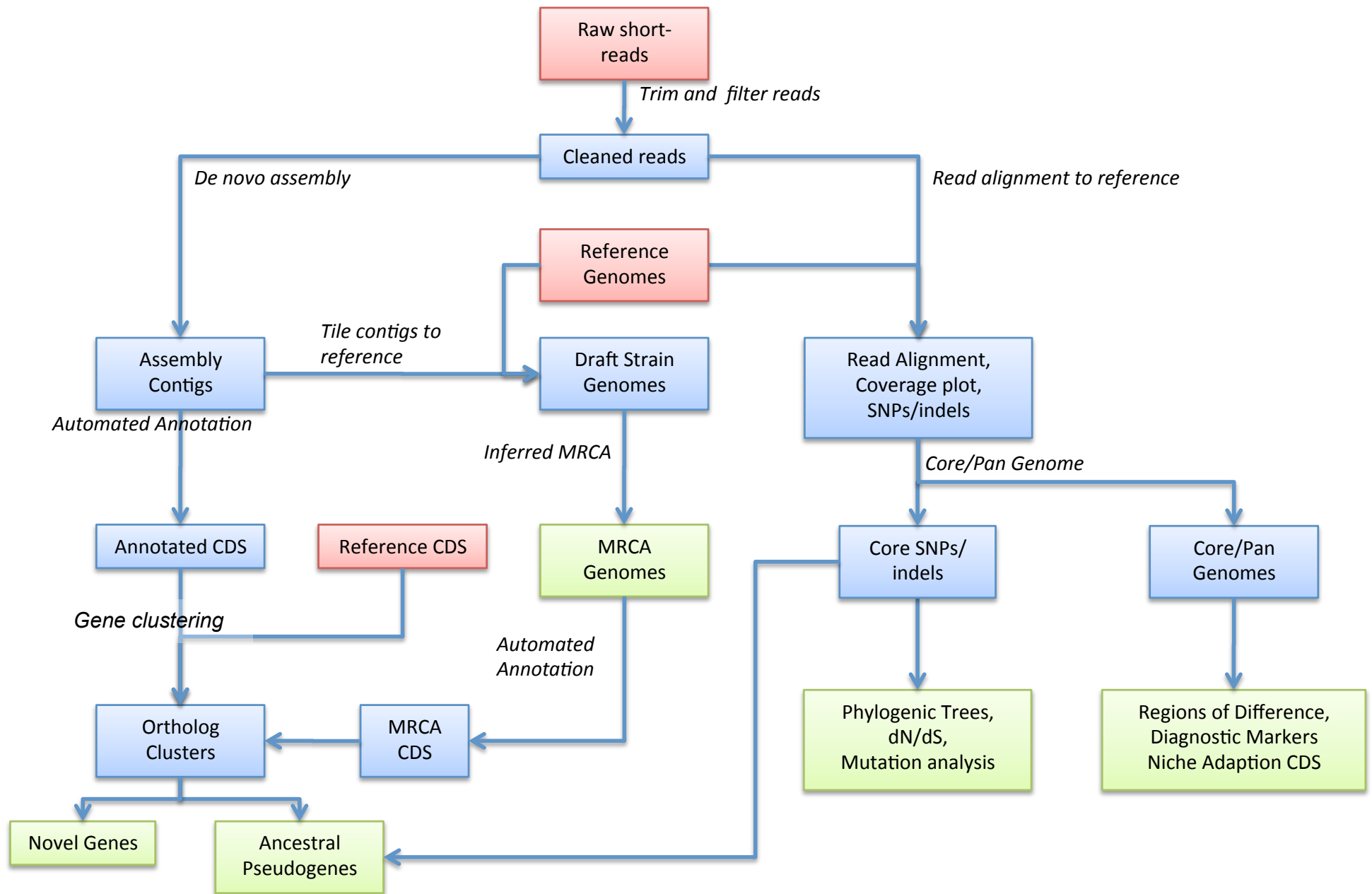

Supplement: Additional file 9: Figure S1 — Work Flow. Schematic of the workflow with intermediate stages of the data shown in blue boxes. The inputs to the process are shown in red and comprise the annotated reference genomes and the short read sequencing data of the study isolates. The results are shown in green and include the phylogeny of the complex, novel CDS within each of the isolates, core and accessory genomes of the complex and putative ancestral pseudogenes. [file 1471-2164-13-258-S9.pdf]
